# Objective assessment of intraoperative skills for robot-assisted partial nephrectomy (RAPN)

**DOI:** 10.1007/s11701-023-01521-1

**Published:** 2023-01-23

**Authors:** Rui Farinha, Alberto Breda, James Porter, Alexandre Mottrie, Ben Van Cleynenbreugel, Jozef Vander Sloten, Angelo Mottaran, Anthony G. Gallagher

**Affiliations:** 1 Orsi Academy, Proefhoevestraat 12, Melle, 9090 Ghent, Belgium; 2 Department of Urology, Onze-Lieve-Vrouw Ziekenhuis, Aalst, Belgium; 3 Department of Urology, São José Hospital, Lisbon, Portugal; 4 Department of Urology, Fundació Puigvert, Universitat Autonoma de Barcelona, Barcelona, Spain; 5 Swedish Urology Group, Swedish Medical Center, Seattle, WA USA; 6 Department of Urology, University Hospitals Leuven, Louvain, Belgium; 7 Department of Development and Regeneration, KU Leuven, Louvain, Belgium; 8 Department of Mechanical Engineering, Section of Biomechanics, KU Leuven, Louvain, Belgium; 9 Division of Urology, IRCCS Azienda Ospedaliero-Universitaris di Bologna, Bologna, Italy; 10 University of Bologna, Bologna, Italy; 11 Faculty of Medicine, KU Leuven, Louvain, Belgium; 12 Faculty of Life and Health Sciences, Ulster University, Derry, Northern Ireland UK

**Keywords:** Surgical training, Robot-assisted partial nephrectomy, Proficiency-based training, Metrics, Construct validation, Renal cancer

## Abstract

RAPN training usually takes place in-vivo and methods vary across countries/institutions. No common system exists to objectively assess trainee capacity to perform RAPN at predetermined performance levels prior to in-vivo practice. The identification of objective performance metrics for RAPN training is a crucial starting point to improve training and surgical outcomes. The authors sought to examine the reliability, construct and discriminative validity of objective intraoperative performance metrics which best characterize the optimal and suboptimal performance of a reference approach for training novice RAPN surgeons. Seven Novice and 9 Experienced RAPN surgeons video recorded one or two independently performed RAPN procedures in the human. The videos were anonymized and two experienced urology surgeons were trained to reliably score RAPN performance, using previously developed metrics. The assessors were blinded to the performing surgeon, hospital and surgeon group. They independently scored surgeon RAPN performance. Novice and Experienced group performance scores were compared for procedure steps completed and errors made. Each group was divided at the median for Total Errors score, and subgroup scores (i.e., Novice HiErrs and LoErrs, Experienced HiErrs and LoErrs) were compared. The mean inter-rater reliability (IRR) for scoring was 0.95 (range 0.84–1). Compared with Novices, Experienced RAPN surgeons made 69% fewer procedural Total Errors. This difference was accentuated when the LoErr Expert RAPN surgeon’s performance was compared with the HiErrs Novice RAPN surgeon’s performance with an observed 170% fewer Total Errors. GEARS showed poor reliability (Mean IRR = 0.44; range 0.0–0.8), for scoring RAPN surgical performance. The RAPN procedure metrics reliably distinguish Novice and Experienced surgeon performances. They further differentiated performance levels within a group with similar experiences. Reliable and valid metrics will underpin quality-assured novice RAPN surgical training.

## Introduction

Robot-assisted partial nephrectomy (RAPN) is well established as a surgical treatment for T1a renal masses [1–3]. Guidelines on how surgeons should perform RAPN usually come from experienced surgeons and vary across institutions/countries [4, 5]. In addition, surgeon and hospital’s RAPN volume directly impact on complication rates [6–10].

The relationship between RAPN approaches and complication rates also informs the way surgeons are trained [9, 11], and increased focus on patient safety compels a paradigm shift in the training methodology. At the start of the twenty-first-century surgeons still acquire the vast majority of their procedure skill on patients. Furthermore, surgical competence is still assessed through process measure (i.e., number of procedures performed or time in training), instead of using meaningful performance metrics [12].

Proficiency-based progression (PBP) training represents a paradigm shift in how surgeons learn new skills, offering objective and validated performance metrics to support and track the progression of their operative skills. It also utilizes a standardized and validated system to objectively evaluate trainee capacity to perform RAPN at a predetermined performance level prior to in-vivo practice on patients [13–16].

Several prospective, randomized and blinded controlled trials have shown that metric-based simulation training to proficiency produces superior surgical skills in comparison with traditional quality-assured training approaches and impact on clinical outcomes [16–22]. This motivated the authors to develop performance metrics for a RAPN procedure which would then underpin a metric-based training program. The identified metrics were subjected to detailed scrutiny and discussion by an international panel of expert RAPN surgeons. The outcome was a clear consensus on the metrics and their operational definitions [23].

This study aims to examine the reliability, construct and discriminative validity of these metrics. Construct validity establishes the extent to which the metrics discriminate between different levels of expertise of RAPN surgeons (i.e., the Experienced group of RAPN surgeons should perform the procedure better than the Novice RAPN surgeons) [24]. Discriminative validity assesses whether the metrics are able to differentiate performance levels within a group of surgeons with similar experience, denoting high sensitivity and specificity [24–26].

## Patients and methods

After obtaining Institutional Review Board Approval, and written informed consent from study participants, the authors compared intraoperative RAPN performance scores of 7 Novices and 9 Expert surgeons. A Novice was defined as having performed ≤ 25 RAPNs, and an Expert as having performed ≥ 250 RAPNs.

### Procedure videos

RAPN videos to be scored only included left-sided renal tumors, located in the lower pole or middle part of the kidney, with < 4 cm diameter and being > 50% exophytic.

### Assessors

Two consultant RAPN surgeons were trained to be assessors, by a behavioral scientist and education-training expert (AGG). Eight hours of face-to-face meetings and online conference calls were conducted using Zoom (San Jose, California, US) [14, 27]. Both assessors studied the methods of PBP metrics for RAPN and Global Evaluative Assessment of Robotic Skills (GEARS) scoring in detail [23, 28]. Multiple unedited videos of RAPN performed by different surgeons of varying degrees of expertise were used to illustrate what to score.

Each assessor then scored the videos independently until IRR [IRR: agreements/(agreements + disagreements)] ≥ 0.8 [14, 29]. Disagreements, conflicts or uncertainty around scoring entailed further discussion with AGG to improve scoring and assessments.

Once both assessors could independently, consistently and reliably (i.e., IRR ≥ 0.8) score operative performance, 24 complete unedited recordings of RAPN, performed by 7 Novice and 9 Experienced surgeons were scored. Assessors were blinded to the identity or level of expertise of the operating surgeon. Each video was evaluated using both binary metrics and GEARS, and scorings were tabulated. An IRR was considered acceptable if it was ≥ 0.8.

### Performance metrics

A procedure Step was defined as a component task, the series aggregate of which constitutes the completion of a specific procedure. An Error was defined as a deviation from optimal performance. A Critical Error was defined as an event or occurrence involving a serious deviation from optimal performance during a procedure that either (1) jeopardizes the success or the desired result of the procedure or (2) creates iatrogenic insult to the patient’s tissues [17].

### Statistical analysis

Assuming approximately equal variance in each group, data were analyzed with a Mixed Model regression analysis. Fixed effect was Group (i.e., Novice and Experienced Groups) and the repeated measure was the Procedure number used to determine if there was a statistical difference for the primary endpoints (number of completed Steps, Errors, Critical Errors, and Total Errors) between the Novice and the Experienced group.

Results are reported in terms of statistical estimate (Est), standard errors (SE), degree of freedom (*df*), test statistic (*t*), and probability value (*p*). It was assessed the statistical difference between the first and second procedure for the surgeons that have submitted a second RAPN.

The median Total Errors score was calculated for the Novices and Experienced surgeons and a dummy dichotomous variable was created (i.e., based on scores above or below the median Total Errors score). A Novice LoErrs (Total Errors score below the median) and a Novice HiErrs (Total Errors score above the median) subgroups were defined. We used the same approach to create Experienced LoErrs and HiErrs subgroups. The IBM Statistical Package for the Social Sciences, version 28 (SPSS®; IBM, Corp., Armonk, NY, USA) was used.

### IRR assessment

RAPN videos were scored for occurrence (i.e., event/ metric unit was observed) by each of the assessors and scores tabulated. The difference and discrepancies between reviewers were compared. Video score IRR was assessed using the formulated Agreements/(Agreements + Disagreements) [14, 15, 30].

## Results

### Binary metrics scores

**Fig. 1:**
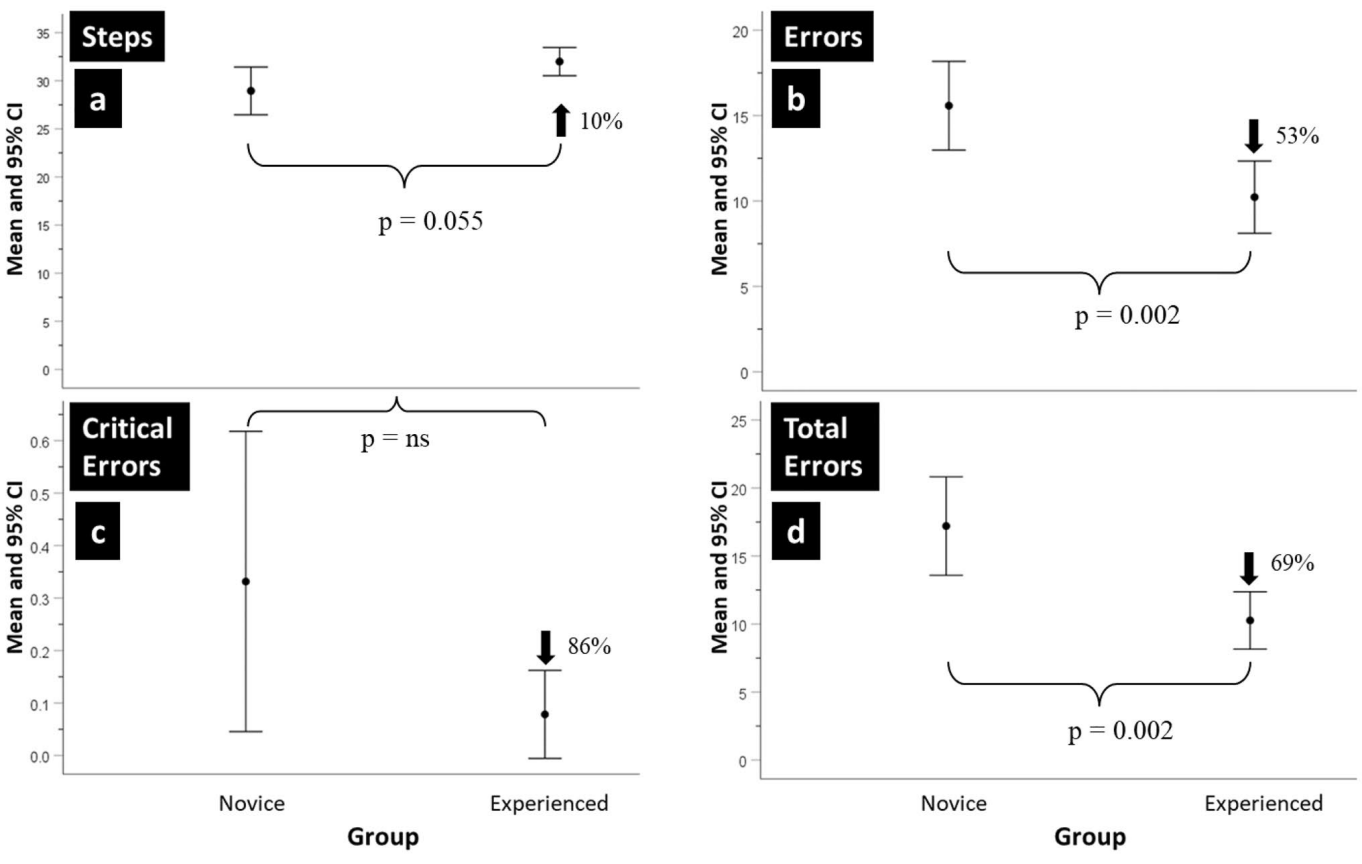
**a–d** Novice and Experts using binary metrics. The mean and 95% confidence intervals (CI) of the number of Steps completed, Errors, Critical Errors and Total Errors made by Novice and Experienced surgeons when performing the robot-assisted partial nephrectomy (RAPN) procedure

The mean IRR was 0.9 (SD = 0.03), and no assessment fell below 0.8. The mean and 95% confidence intervals (CI) for the number of procedure Steps, Errors, Critical Errors, and Total Errors (sum of Errors and Critical Errors) made by the Novice and Experienced surgeons are shown in Figs. 1a–d.

The Novice group on average, completed 10% (i.e., 2) fewer procedure Steps than the Experienced group, but this difference was not statistically significant. The later made 53% fewer procedure Errors than the former, being this difference statistically significant (Est = 5.46, SE = 1.58, *df* = 21.968, *t* = 3.454, *p* = 0.002). Overall, the Critical Errors rate was low. Although the Novice group made more Critical Errors and showed greater score variability, the difference was not statistically significant. On average, the Novice group made 69% more Total Errors than the Experienced group, which was found to be statistically significant (Est = 6.694, SE = 1.92, *df* = 21.344, *t* = 3.478, *p* = 0.002). Procedure numbers (i.e., Procedure 1 and 2) had no significant impact on the statistical results.

Performance data for each group was divided at the median of the Total Errors score, to create two sub-groups. The median Total Errors score for the Experienced surgeons was 10.25. Therefore, Experienced surgeons who made more than 10.25 Total Errors were classified as surgeons above the median who had a high error rate (Experienced HiErrs; *n* = 5), and who made less than 10.25 Total Errors were classified as surgeons below the median who had a low error rate (Experienced LoErrs, *n* = 7). The same approach was used for the Novice surgeons. The median Total Errors score for the Novice surgeons was 17.25. Novice surgeons who made more than 17.25 Total Errors were classified as surgeons above the median who had a high error rate (Novice HiErrs; *n* = 6), and who made less than 17.25 Total Errors were classified as surgeons below the median who had a low error rate (Novice LoErrs, *n* = 6).

**Fig. 2:**
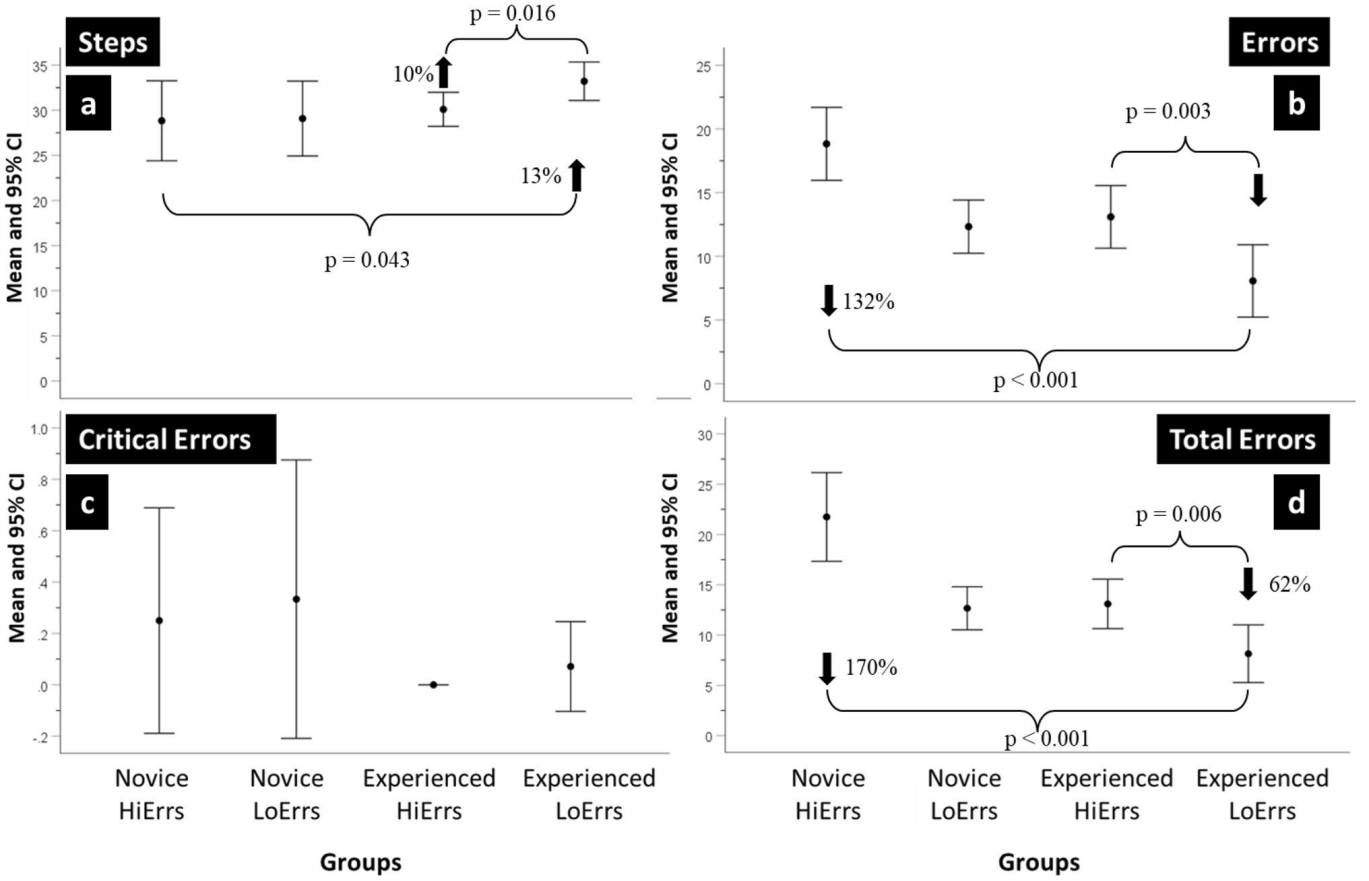
**a–d** HiErrs and LoErrs using binary metrics. The mean and 95% confidence intervals (CI) of the number of Steps completed, Errors, Critical Errors and Total Errors made by Novice and Experienced surgeons in the ‘high’ and ‘low’ errors groups when performing the robot-assisted partial nephrectomy (RAPN) procedure

A summary of the performances of the resulting four groups is shown in Fig. 2a–d for procedure Steps, Errors, Critical Errors and Total Errors. Differences between groups for procedure Steps were assessed for statistical significance with a Mixed Model regression analysis where the fixed effects were Group (i.e., Novice HiErrs and LoErrs and Experienced HiErrs and LoErrs) and the repeated measure was the Procedure number (i.e., Procedure 1or Procedure 2).

Although differences between the subgroups in the number of procedure Steps completed were small, it was found that the Experienced LoErrs completed significantly more procedure Steps than the Novice HiErrs (Est = − 3.897, SE = 1.702, *df* = 18.173, *t* = 2.172, *p* = 0.043) and the Experienced LoErrs completed more procedure Steps than the Experienced HiErrs although this difference was not statistically significant.

Differences were observed for the procedure Errors. The Experienced LoErrs made 132% fewer Errors than the Novice HiErrs, being this difference statistically significant (Estimate = 10.971, Standard Error = 1.422, *df* = 19.975, *t* = 7.718, *p* < 0.001) and 62% fewer Errors than the Experienced HiErrs (Estimate = 5.007, Standard Error = 1.421, *df* = 16.089, *t* = 3.542, *p* = 0.003). In contrast, the Experienced HiErrs made a similar number of procedure Errors as the Novice LoErrs.

Overall, the Critical Error rate was low. Both Novice HiErrs and LoErrs made the most Critical Errors and demonstrated the greatest variability. The Experienced HiErrs made no Critical Errors and the Experienced LoErrs made on average 0.07, although this difference was not statistically significant.

Concerning Total Errors, the Experienced LoErrs made significantly fewer Total Errors than the Novice HiErrs (Est = 13.577, SE = 1.618, *df* = 19.110, *t* = 8.389, *p* < 0.001) and the Experienced HiErrs (Est = 4.984, SE = 1.524, *df* = 13.180, *t* = 3.247, *p* = 0.006). The Experienced HiErrs made a similar number of Total Errors to the Novice LoErrs. In this analysis of procedure Steps, Errors, Critical Errors, and Total Errors, there were also no significant main or interaction effects of procedure number.

### GEARS score

**Fig. 3:**
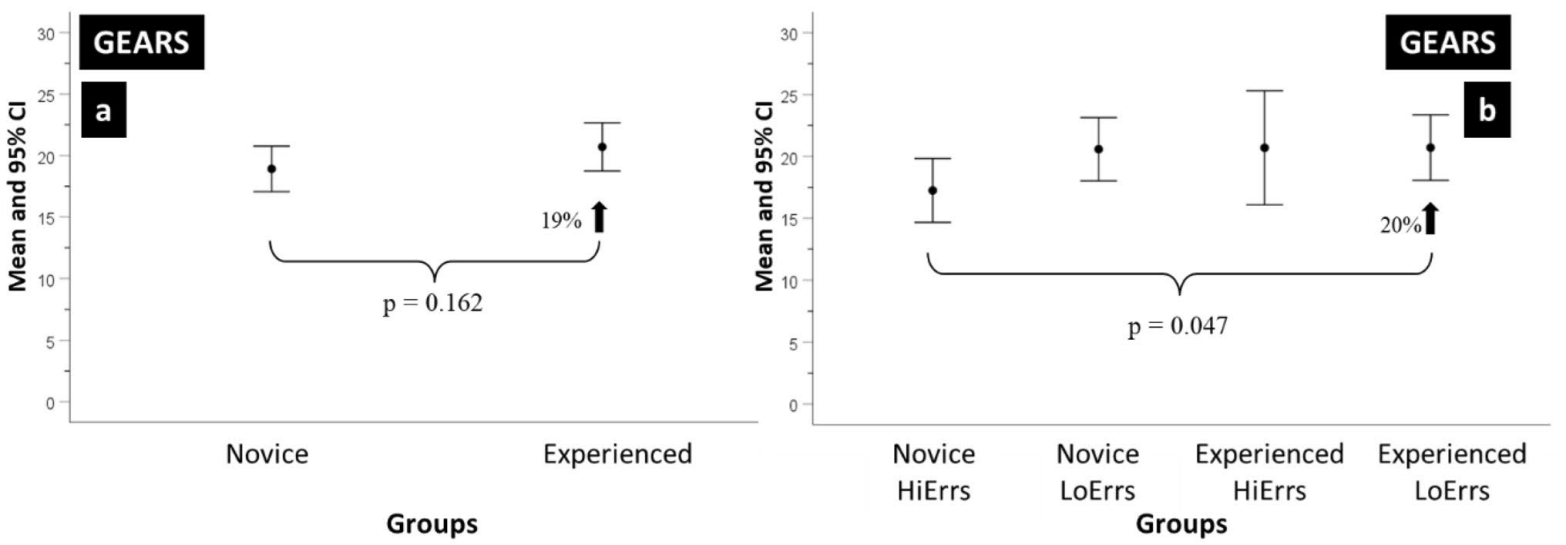
**a**, **b** HiErrs and LoErrs using GEARS. The mean and 95% confidence intervals (CI) of the number GEARS scores **a** Novice and Experienced surgeons and **b** Novice and Experienced surgeons in the ‘high’ and ‘low’ errors groups when performing the robot-assisted partial nephrectomy (RAPN) procedure

Figures 3a and b show the mean and 95% CI score of operative performance using the GEARS assessment instrument. The mean IRR for GEARS was 0.44 (0–0.8).

Figure 3a shows the comparison between Experienced and Novice groups. The former had a 19% higher score than the later, and this difference was not statistically significant.

Both groups were divided into LoErrs and HiErrs subgroups following the approach previously described. Figure 3b shows that the Experienced LoErrs score was 20% higher than the Novice HiErrs and this difference was statistically significant (Est = − 3.344, SE = 1.57, *df* = 18.228, *t* = − 2.13, *p* = 0.047).

None of the other score differences were statistically significant, and although the Experienced HiErrs scored similar to the Experienced LoErrs, they had greater score variability as demonstrated by the larger CI.

### Receiver operating characteristic (ROC) analysis

We evaluated the capacity of the five assessments (i.e., Steps, procedure Errors, Critical Errors, Total Errors, and GEARS score) to discriminate the Experienced LoErrs performance in comparison to the other subgroups at a sensitivity threshold of 0.8 and 0.9.

**Fig. 4:**
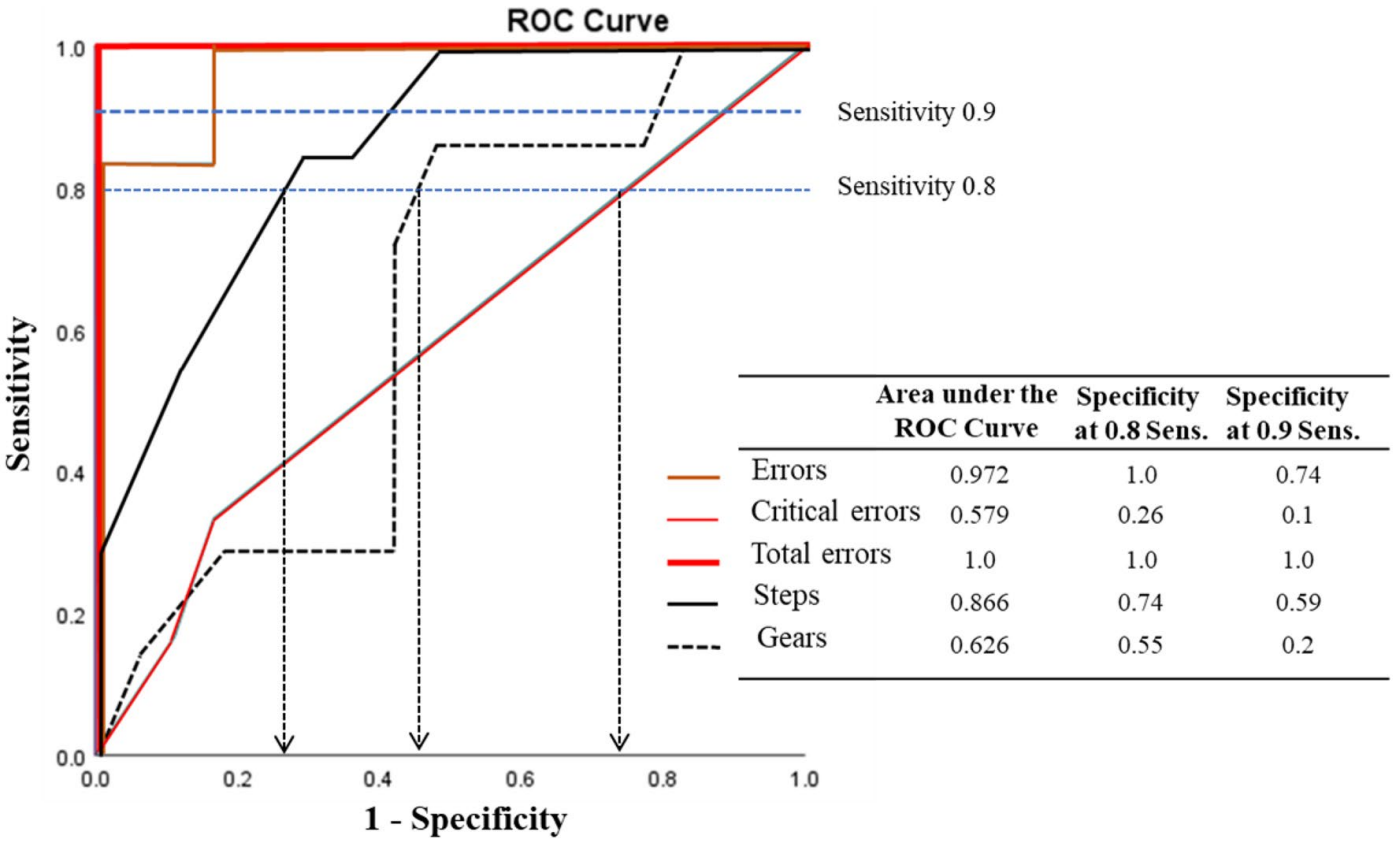
ROC curve. The Receiver operator characteristic (ROC) Area Under the Curve showing the Checklist (i.e., Procedure Steps, Errors, Critical Errors and Total Errors) and GEARS Assessment discrimination (i.e., Specificity) levels for surgeon groups as Sensitivity thresholds were varied (i.e., 0.8 Sensitivity and 0.9 Sensitivity levels)

The Area Under the Curve (AUC) for the Critical Errors was the lowest (i.e., AUC = 0.579) and the Total Errors AUC was the highest (i.e., AUC = 1.0) (Fig. 4).

The interpolation on the Specificity of a Sensitivity level of 0.8 and 0.9 was also analyzed. At a Sensitivity level of 0.8, the Specificity was 1.0 for Total Errors, 0.74 for procedure Errors, 0.55 for GEARS and 0.26 for Critical Errors. Only Total Errors demonstrated an excellent level of Specificity at a Sensitivity of 0.9 since all other measures had a Specificity level < 0.8 (Fig. 4).

## Discussion

The increasing use of RAPN necessitates the requirement for better training and probably the establishment of a standardized PBP training program with the goal to improve surgical outcomes [31, 32]. Implementation of better training will allow the objective, transparent and fair assessment of surgical skills. A Delphi meeting with experienced surgeons, established the face and content validity of the RAPN metrics used in this study [23]. We then sought to establish evidence supporting the construct validity of the corresponding metrics.

We demonstrated that RAPN metrics could be scored reliably with an IRR > 0.8 and that the metrics reliably discriminated between Experienced and Novice RAPN surgeons. The metrics which discriminate best were the number of Errors and Total Errors. The Experienced group made 53% fewer objectively assessed procedure Errors than the Novice group, and the later made 69% more Total Errors than the former. We then implemented a methodology previously described [33–38], which partitioned each of the two groups at their median Total Errors score (i.e., LoErrs and HiErrs).

After delineating the four surgical performance subgroups (Experienced LowErrs, Experience HiErrs, Novice LowErrs and Novice HiErrs) key findings emerged. This novel approach to the analysis of operative performance showed a greater capacity to differentiate objectively assessed surgical performance. The Experienced LowErrs made between 132 and 170% fewer Errors and Total Errors, respectively, than the Novice HiErrs. As reported in previous studies it also became clear that surgical experience and seniority did not always translate into better surgical performance [39]. We found that the Experienced LoErrs group made 62% fewer Total Errors than Experienced HiErrs and that the Experienced HiErrs performed at the same level as the Novice LowErrs.

It might be argued that objective assessment of intraoperative performance on one occasion is a poor indicator of surgical skill, but published evidence has already shown that peer-assessed surgical skills strongly predict clinical outcomes [33, 36].

The results of GEARS assessments demonstrated weak levels of IRR and were lower than the IRR levels observed when using RAPN binary metrics (0.44 vs 0.9). GEARS assessment also struggled to differentiate the objectively assessed performance of Experienced and Novice surgeons.

This is the first report using metrics that objectively characterize intraoperative RAPN performance, reporting quantitative evidence to support construct and discriminative validity.

These results provide a stepping stone for the construction of a standardized RAPN training program following a PBP methodology. Specifically, they provide the tool to establish performance benchmarks (i.e., proficiency levels), that trainees must demonstrate before training progression. Trainees would only be allowed to operate on the patient after demonstrating the ability to perform to the quantitatively defined performance level. They can (i) have as many training trials as they wish, (ii) be supported by faculty who know and can score the metrics reliably and iii) know how to use the metrics for deliberate rather than repeated practice [40]. Therefore, the trainee is offered metric-based training that is objective, transparent and fair and produces performances that have been shown to be ~ 60% better than traditional training courses [41]. That said, he does not progress to performing the procedure on patients until demonstrating the requisite benchmarks [13, 15, 42].

Our study found that the results were not supportive of the reliability and validity of GEARS scoring on RAPN performance, adding to the accumulating evidence that Lickert scales are not robust enough to score surgical performance.

### Study limitations

- The number of RAPN videos evaluated might limit the generalizability of our analysis and any firm conclusions about performance variability by very experienced operators. There is, however, a steady stream of reports on very experienced surgeons that perform poorly on straightforward and familiar tasks when their performance is objectively assessed by reviewers blinded to experience level [35, 37, 38, 43, 44];
- The RAPN metrics used in this study are only applicable to left-sided transperitoneal RAPN. Right-sided and retroperitoneal approaches, cannot be scored using these metrics, although the large majority of evidence on RAPN techniques and outcomes refer to the transperitoneal approach;
- These metrics were developed for straightforward cases and patient characteristics (i.e., age, body mass index, previous abdominal surgery or other comorbidity indexes) were not taken into account which may have influenced the differences in performance. However, if a trainee cannot do a straightforward case, they probably should not be performing a more complex one;
- Although *Novice* surgeons were required to complete the RAPN independently, we cannot exclude a marginal bias derived from the impact of clinical supervision from an *Experienced* surgeon. This, however, would mean that the differences observed in this study are an underestimation of the real differences.

## Conclusions

We report RAPN metrics that reliably and consistently discriminate the intraoperative performance of Expert and Novice Surgeons. The number of Total Errors demonstrated the highest level of specificity and sensitivity in this discrimination. GEARS demonstrated poor reliability in scoring RAPN surgical performance. These metrics lay the foundation to implement a simulation-based PBP training program.

### Patient summary

We developed metrics for scoring RAPN performance. Two experienced surgeons, trained to use RAPN metrics, scored anonymized video recorded RAPN performance of novice and expert surgeons. Our study showed that the RAPN metrics consistently and reliably identify “true” Experts and “true” Novices.

## Data Availability

All data were obtained from the experiments conducted by the authors.

## Abbreviations

RAPN: Robot-assisted partial nephrectomy
IRR: Inter-rater reliability
PBP: Proficiency-based progression
AGG: Anthony gallagher
GEARS: Global evaluative assessment of robotic skills
Est: Statistical estimate
SE: Standard errors
*df*: Degree of freedom
*t*: Test statistic
*p*: Probability value
CI: Confidence interval
SD: Standard deviation
ROC: Receiver operating characteristic
AUC: Area under the curve
